# Learning experience of domestic and international students in blended courses: An ecological perspective

**DOI:** 10.1371/journal.pone.0285389

**Published:** 2023-05-12

**Authors:** Feifei Han

**Affiliations:** 1 Institute for Learning Sciences and Teacher Education, Australia Catholic University, Brisbane, Australia; 2 Department of Pedagogy and Psychology, Faculty of Education, University of Hradec Králové, Hradec Králové, Czech Republic; Alexandria University Faculty of Nursing, EGYPT

## Abstract

Adopting an ecological perspective on student learning, this study compares the cognitive, social, and material elements of the learning experience of 193 domestic and 120 international students in blended courses. For cognitive elements, domestic students adopted relatively more deep approaches to learning while international students held more positive perceptions towards the blended learning environment. In terms of the social elements, domestic group had a higher proportion of students who chose to collaborate than international group. The domestic and international students also differed with regard to their experience of material elements that international students were more likely to select the option of showing the answer for solving multiple choice questions. International students particularly need help in the improvement of their collaborative learning experience, which may be enhanced by strategies, such as mixing domestic and international students in group work and training international students’ intercultural communication strategies.

## 1. Introduction

According to the Organization for Economic Cooperation and Development (OECD) [1], there were more than five million students enrolled in tertiary education outside their country of citizenship worldwide. Ranked the second, Australia attracts international students from all over the world with the promise of a world-class education, accounting for 8.0% of the global education market share [1]. In May 2021, there were 525,892 international students enrolled in education programs in Australia, with 54.0% in the higher education sector [2].

Bista and Foster has suggested that international students face diverse difficulties and challenges in transition to new learning environments, including homesickness, social isolation, weak language abilities, different educational systems, cultural and value shock [3]. The ongoing advances in the internet and computer technology have created new challenges for international students when they are involved in blended courses. Such courses require students to move constantly forth and back between physical and virtual spaces; to communicate and interact with peers, teachers, and learning resources both face-to-face and online; and to actively seek to understand how different course components are related to and complement each other [4].

While international students’ learning experiences in blended courses have been explored to some extent [5–8], little is known about how their experiences compared with those of domestic students. This gap in the literature has motivated the design of this study, which compares domestic and international university students’ learning experience in blended courses. Such comparison will reveal how the needs of one group can be more deeply understood by considering the outcomes of the other and will enable international students to enhance their learning experiences of university learning and teaching.

### 1.1 The learning experiences of domestic and international students in blended courses

Widely adopted in higher education worldwide, blended courses are “a systematic combination of co-present (face-to-face) interactions and technologically-mediated interactions between students, teachers and learning resources” [9]. Although a couple of studies have compared domestic and international students’ learning experience in blended courses, inconsistent results have been reported [10, 11].

In a recent study, Dang et al. [10] compared 639 domestic students’ and 60 international students’ perceptions of blended courses using a survey. The students were enrolled in a first-year computer information course at a public university in the United States. They found that in general international students had more positive learning experience than domestic students in learning in blended courses. Their survey examined students’ perceptions of their blended courses across five dimensions: individual, social, technology, adoption, and impact. The survey also asked students to rate their actual involvement in the blended courses, their intention to enroll in other blended courses in the future, and their expectation of their academic performance.

The only aspect that domestic students held higher-level positive perceptions than international counterparts was the Internet self-efficacy (an aspect of the individual dimension). The international students, however, were more positive than their domestic peers in a number of aspects across all the five dimensions except for the social dimension. In the individual dimension, international students had a higher-level motivation to learn in blended courses, and were more cognitively engaged during the learning process. Of the technology dimension, international students felt relatively more positive about how the learning activities and tasks were mapped into the online learning system. In the adoption dimension, international students reported more enjoyment, less frustration, and a more positive attitude. The international students also reported higher ratings on their actual use of the online learning system in the blended courses, on their intention to take more blended courses in the future, and on their anticipated academic performance in the blended courses.

The generally more positive experience of international students over domestic students in the Dang et al.’s study [10], however, contrasts with the results of Chew’s study [11]. Chew compared the perceptions of the support of the online part of the learning in different blended courses between 41 Malaysian students enrolled in an Australian university and 35 Australian local students using a mixed-method approach. The quantitative outcomes of the study did not find any significant difference between domestic and international students’ responses on all the fours scales of the support of the online learning (computer usage, lecturer support, students’ interaction and collaboration, and personal relevance).

However, the qualitative data of the open-ended questionnaire revealed that Malaysian students considered the layout of the online discussion forum was poorly designed and the online discussions were difficult and boring. They also reported a preference for face-to-face discussions. Chew attributed the results to students’ lack of experience with the online learning as the participants were only in their first two years of the university studies [11]. It should be noted that Chew’s study only focused on the online part in blended courses, and hence did not reflect the complete picture of the students’ learning experience of the blended courses. Given the dearth of research in this area and the inconsistent findings, clearly more empirical investigations are required on the learning experience of domestic and international students in blended courses.

### 1.2 Ecological perspectives on the learning experiences of university students in blended courses

In blended courses, students are increasingly involved in decision-making in the learning processes, such as where to learn (e.g., going to the library or researching at home), how to learn (e.g., interacting with lecture content online or conducting laboratory experiments), and with whom to learn (e.g., by themselves, in pairs, or in groups). Just a short reflection on these questions for modern experience of university learning reveals that the learning experiences in blended courses design are made up of multiple elements, which can be usefully divided into those related to students’ cognition (e.g., approaches and perceptions) [12]; their social interactions in learning (e.g., collaborative learning) [13]; and their engagement with the material elements in the physical and virtual learning spaces (e.g., interactions with learning activities) [14]. Each decision made by students in the cognitive, social, and material elements of their course can be considered as part of the whole learning experiences.

To better understand the complexity of students’ learning experiences in blended courses, this study adopted an ecological perspective, which offers a more holistic picture of learning in authentic and contextualized settings through multiple methodologies and sources of data [4]; and examined the learning experiences of domestic and international students across the cognitive, social and material elements. Consequently, the multiple methodologies used in this study were drawn from three areas.

One substantial body of work–student approaches to learning (SAL) research, demonstrates that how students’ understandings and perceptions of learning, and their decisions of the intent and strategies in learning, are consistently associated with their academic achievement [12]. Another body of research–social network research in education uses social network analysis (SNA) to examine various types of students’ networks (e.g., collaborative networks, friendship networks, discussion networks), and the relations between different patterns of these networks and students’ academic learning outcomes [15]. A third field–learning analytics research uses learning trace data and educational data mining techniques to provides observable evidence of what student actually do in the learning process when they interact with technologies [16]. The observational methods and data are able to offer a type of triangulated evidence beyond relatively subjective data from self-report instruments [4].

The methodologies from these three areas share similarities as they all use individual student as the unit of the analysis. At the same time, they also complement each other, each measuring aspects in the learning experience which the others are not designed to. Hence, combining methodologies from research in SAL, social network research in education, and learning analytics allows the cognitive, social, and material elements of students’ learning experience to be examined respectively and simultaneously [17]. The following sections review the methodologies and the relevant prior research in the areas of SAL, SNA, and learning analytics.

### 1.3 Student approaches to learning (SAL) research

Methodologies from SAL research identify variations in the students’ experience of learning [12], in particular various elements in the cognitive aspects (i.e., learners’ internal states) of such experience. Key constructs in this research have shown that the way students approach their learning (e.g., deep and surface approaches) and the way they perceive their learning context and environment (e.g., positive and negative perceptions) are significantly associated with each other and with students’ academic achievement [18].

Consistent findings are reported that surface approaches to learning, which are characterized by mechanistic procedures, seeking to produce formulaic responses, and being not engaged with the ideas and conceptions in learning, tend to be related to negative perceptions of the learning context, in which the quality of teaching is often perceived as poor, the goals as unclear, the assessment as irrelevant, and the satisfaction low [12]. The deep approaches, which enable students to engage meaningfully with the subject matter, and motivate them to experiment and make decisions about using the most appropriate learning strategies in their studies, are likely to be associated with positive perceptions of the learning environment, in which satisfaction and the quality of teaching are perceived as high, workload and assessment tasks are considered being appropriate [19].

Primarily drawing on the self-report data, methodologies in SAL research are suitable to examine learners’ cognitions. However, such methodologies are not robust to provide effective measurements of collaboration in learning and fails to capture what students actually do rather than what they report they do. Such limitations are addressed in our study by complementary methodologies in SNA and in learning analytics research.

### 1.4 Social network research in education

Social network research in education draws on SNA methodologies, which identify, detect, and interpret roles of individuals in a group and patterns of ties amongst them [20]. SNA uses graph theory to visualize various kinds of networks and to provide mathematical measures to describe individuals’ positions in the network and their relations [21].

In educational contexts, past studies have examined different types of networks amongst students, such as social and friendship networks [22], knowledge sharing networks [23], networks of the interaction between students and teachers [24], online discussion networks [25], study partner networks [26], and student collaboration networks [27]. In this study, SNA is used to examine the patterns of students’ collaboration, which can shed some light on the social elements of students’ learning experience in blended courses.

### 1.5 Learning analytics research

Learning analytics research has emerged in the last couple of decades stimulated by the integration of information communication technologies into learning [28]. The large amount of digital trace data recorded by technologies can be used profitably to describe students’ learning actions. Learning analytic research has been conducted to detect at-risk students, identify learning strategies, predict attrition, monitor student affect, provide feedback, advise career plans, and explain learning achievement [29, 30].

The current study uses methodologies in learning analytics research to capture measurements of student’ online interactions, which shed some light on material aspects in students’ learning experience in blended learning contexts. While claiming objectivity, learning analytics research is often limited in its potential to uncover the underlying intentions involved in learning as this area of research is yet to systematically include methods that reveal the ‘why questions’ underpinning students’ learning actions. To improve insights of domestic and international students’ learning experiences in blended courses, this study combines self-report methods used in SAL and SNA and observational methods used in learning analytics research.

The overarching research question addressed in the study is: to what extent do Australian domestic and international students differ in their learning experiences in blended courses? The overarching research question can be divided into the following supporting questions:

- To what extent do Australian domestic and international students differ in the cognitive elements of learning experience in blended courses?
- To what extent do Australian domestic and international students differ in the social elements of learning experience in blended courses?
- To what extent do Australian domestic and international students differ in the material elements of learning experience in blended courses?

## 2. Materials and methods

### 2.1 Sampling and data collection procedure

The research adopted a convenience sampling method, which was due to the need to access to the online course site in a bespoke learning management system (LMS). The blended course was a computer science course in School of Computer Science and Information Technologies Upon the approval of the study by The Human Research Ethics Committee of the researcher’s University, all the students enrolled in the course were invited to voluntarily take part in the study. They were given a Participant Information Statement and Participant Consent Form, which explained that participation required signing a written consent form and completing a close-ended and an open-ended questionnaire. They were also asked to give permissions to access to their learning activities in LMS and the course marks should they participate. Students were given one week to decide if they would like to participate or not. Finally, 193 domestic students and 120 international students enrolled in the course participated in the study.

The data collection was undertaken towards the end of the semester before the completion of the course. This ensured that the participants had relatively comprehensive learning experience of the course to reflect upon.

### 2.2 Description of the blended course

The blended course lasted a full semester. It consisted of a face-to-face part and a self-paced online part. The face-to-face part consisted of weekly two-hour lectures, weekly two-hour tutorials, and weekly three-hour laboratory sessions. Being held in a bespoke LMS, the online part required students to interact with a variety of online learning activities (The details of the activities were provided in the instruments section).

The aims of the course were two-fold: to develop students’ disciplinary knowledge and to develop their graduate skills, which prepare students ready for the work-force employment. In particular, the generic skills of inquiry and collaborations were the two important targeted skills in the course. Strategies for developing students’ inquiry and collaboration skills were embedded in the design of the assessment tasks, in which students were encouraged to work with others to complete a laboratory project, to write a scientific report of the project development, and to co-present the project in an oral presentation.

### 2.3 Instruments

#### 2.3.1 The self-reported Likert-scale questionnaire

The cognitive elements of the learning experience were measured by a valid and reliable Likert-scale questionnaire [31], which was developed using the SAL literature and previous SAL questionnaires [32, 33]. The questionnaire consisted of six scales with anchors of 1 representing “strongly disagree” and 5 indicating “strongly agree”. Two scales measured approaches to learning through inquiry: deep (4 items, α = .67) and surface approaches (7 items, α = .70). Two scales examined approaches to using online learning technologies: deep (7 items, α = .80) and surface approaches (4 items, α = .75). Two scales assessed perceptions of the blended learning environment: perceptions of the integrated learning environment: (4 items, α = .77), and perceptions of the online contributions (6 items, α = .87). The values of Cronbach’s alpha showed accepted reliability.

#### 2.3.2 The self-reported SNA questionnaire

The social elements of the learning experience were measured by a self-reported SNA questionnaire. The questionnaire used the format commonly used in social network research [15]. The questionnaire asked students to write down up to three collaborators in the course according to the frequency of collaborations, and to choose the primary mode of the collaboration from face-to-face or blended.

The most frequent collaborator face-to-face blended

The 2^nd^ most frequent collaborator face-to-face blended

The 3^rd^ most frequent collaborator face-to-face blended

#### 2.3.3 The learning analytic tools in the LMS

The data of the material elements of the learning experience were collected using the learning analytic tools in the LMS, which recorded the frequencies of students’ interactions with different online learning activities specified below:

- dashboard: providing feedback on students’ practice and progression through the course;
- course readings: providing compulsory and supplementary course materials, including files, links to webpages, and course notes, which were arranged by the topics covered in the course;
- videos: enabling students to engage with the video contents in the course as well as laboratory procedure and instructions for developing projects. The interactions with videos were recorded as the proportions of the three sub-categories (i.e., play, pause, and reload) to the total frequencies of videos.
- multiple choice questions: evaluating students’ understanding of concepts in the course. The interactions with multiple choice questions were also recorded as the proportions of the three sub-categories (i.e., correctly answered responses, incorrectly answered responses, and showing the answer) to the total frequencies of multiple choice questions.
- multiple choice questions in videos: assessing students’ abilities to solve practical problems by having questions built in mini-case studies delivered in videos. Similar to the multiple choice questions, students’ interactions were expressed as the proportions of the three sub-categories (i.e., correctly answered responses, incorrectly answered responses, and showing the answer) to the total frequencies of multiple choice questions in videos.

#### 2.3.4 Students’ academic achievement

Students’ academic achievement in the course were the total course marks (maximum = 100) and the scores of each assessment: lecture preparation (20%); laboratory project (30%), which consisted of the quality of the project, the scientific report, and the oral presentation of the project; and the final closed-book examination (50%).

### 2.4 Research design and data analysis methods

The research was designed as a quantitative study, which combined using methods commonly used in SAL research and SNA research. To compare cognitive elements of the learning experience between domestic and international students, the means of the self-reported scales and scores of the academic achievement were compared using one-way ANOVAs.

To compare the social elements of the learning experience between domestic and international students, SNA was applied. SNA generated the proportions of different types of collaborators (i.e., alone, only initiating collaborations, only being nominated as collaborators, and both initiating and being nominated) amongst domestic and international students, which were then compared using two-sample *z*-tests of proportions. SNA also produced a number of centrality measures, namely degree (the extent of collaborations), in-degree (the extent of receiving collaborations), out-degree (the extent of initiating collaborations), closeness (the sum of steps to reach collaborators), betweenness (capacity to gather information based on the student’s position in the network), eigenvector (quality of collaborations of the collaborators they directly connected with), and local clustering coefficient (tendency of students to form closely knitted groups in collaborations). The centrality measures of domestic and international students were compared using one-way ANOVAs.

To compare the material elements of the learning experience between domestic and international students, the frequencies of the students’ interactions with online learning activities and the proportions of sub-categories of the learning activities were analysed using one-way ANOVAs.

All the statistical analyses were conducted in Statistical Package for the Social Sciences 28 and all the SNA was conducted in Gephi 9.2.

## 3. Results

### 3.1 Demography of the participants

Of 313 participants, 193 were domestic students and 120 were international students, accounting for 61.7% and 38.3% respectively. Most of the international students were from Asian countries, with the top five countries being China, India, Nepal, Vietnam, and Malaysia. Although the ranges of age between domestic (between 18 and 31 years old) and international students (between 17 and 23 years) were different. The mean age of the two groups were similar: domestic students: *M* = 19.89, *SD* = 2.38, and international students: *M* = 19.26, *SD* = 1.17.

### 3.2 Comparison of the cognitive elements of the learning experience and academic achievement between domestic and international students (research question 1)

Table 1 presents the results of the one-way ANOVAs on self-reported cognitive elements and on academic achievement. The results show that except for the deep approaches to using online learning technologies scale: *F*(1, 311) = 0.62, *p* = .43, η^2^ = .00), domestic and international students differed significantly on all the self-reported cognitive elements: deep approaches to learning through inquiry: *F* (1, 311) = 4.43, *p* < .05, η^2^ = .01; surface approaches to learning through inquiry: *F* (1, 311) = 10.77, *p* < .01, η^2^ = .03; surface approaches to using online learning technologies: *F* (1, 311) = 13.25, *p* < .01, η^2^ = .04; perceptions of the integrated learning environment: *F* (1, 311) = 5.47, *p* < .05, η^2^ = .02; and perceptions of the online contributions: *F* (1,311) = 25.69, *p* < .01, η^2^ = .08). To be specific, domestic students reported adopting significantly more deep approaches to learning through inquiry, less surface approaches to learning through inquiry, and less surface approaches to using online learning technologies, than international students. However, international students were more positive on both perceptions of the integrated learning environment and perceptions of the online contributions than domestic students.

**Table 1:** Comparison between domestic and international students regarding their cognitive elements and academic achievement.

Variables	Domestic (*n* = 193)		International (*n* = 120)		*F*	*p*	η^2^
	*M*	*SD*	*M*	*SD*			
DAI	3.56	0.66	3.40	0.61	4.43	**.04**	.01
SAI	2.72	0.58	2.95	0.64	10.77	**.00**	.03
DAT	3.66	0.63	3.72	0.56	0.62	.43	.00
SAT	2.43	0.86	2.79	0.83	13.25	**.00**	.04
INTER	3.61	0.80	3.81	0.62	5.47	**.02**	.02
POC	2.97	0.97	3.48	0.67	25.69	**.00**	.08
Total marks	84.33	20.60	79.92	20.07	3.46	.06	.01
Preparation	16.70	4.03	17.67	3.21	5.01	**.03**	.02
Project	25.21	3.75	24.33	4.75	3.29	.07	.01
Final examination	42.42	18.26	37.92	17.90	4.58	**.03**	.02

*Notes*: DAI = deep approaches to learning through inquiry, SAI = surface approaches to learning through inquiry, DAT = deep approaches to using online learning technologies, SAT = surface approaches to using online learning technologies, INTER = perceptions of the integrated learning environment, and POC = perceptions of the online contributions.

As to the academic achievement, domestic and international students did not differ on the total course marks: *F* (1, 311) = 3.46, *p* = .06, η^2^ = .01; and the marks on the research project: *F* (1, 311) = 3.29, *p* = .07, η^2^ = .01; but they scored significantly differently on the class preparation: *F* (1, 311) = 5.01, *p* < .05, η^2^ = .02; and the final examination: *F* (1, 311) = 4.58, *p* < .05, η^2^ = .02. Domestic students obtained higher scores on the final examination but received lower scores on class preparation than international students.

### 3.3 Comparison of the social elements of the learning experience between domestic and international students (results for research question 2)

**Fig 1:**
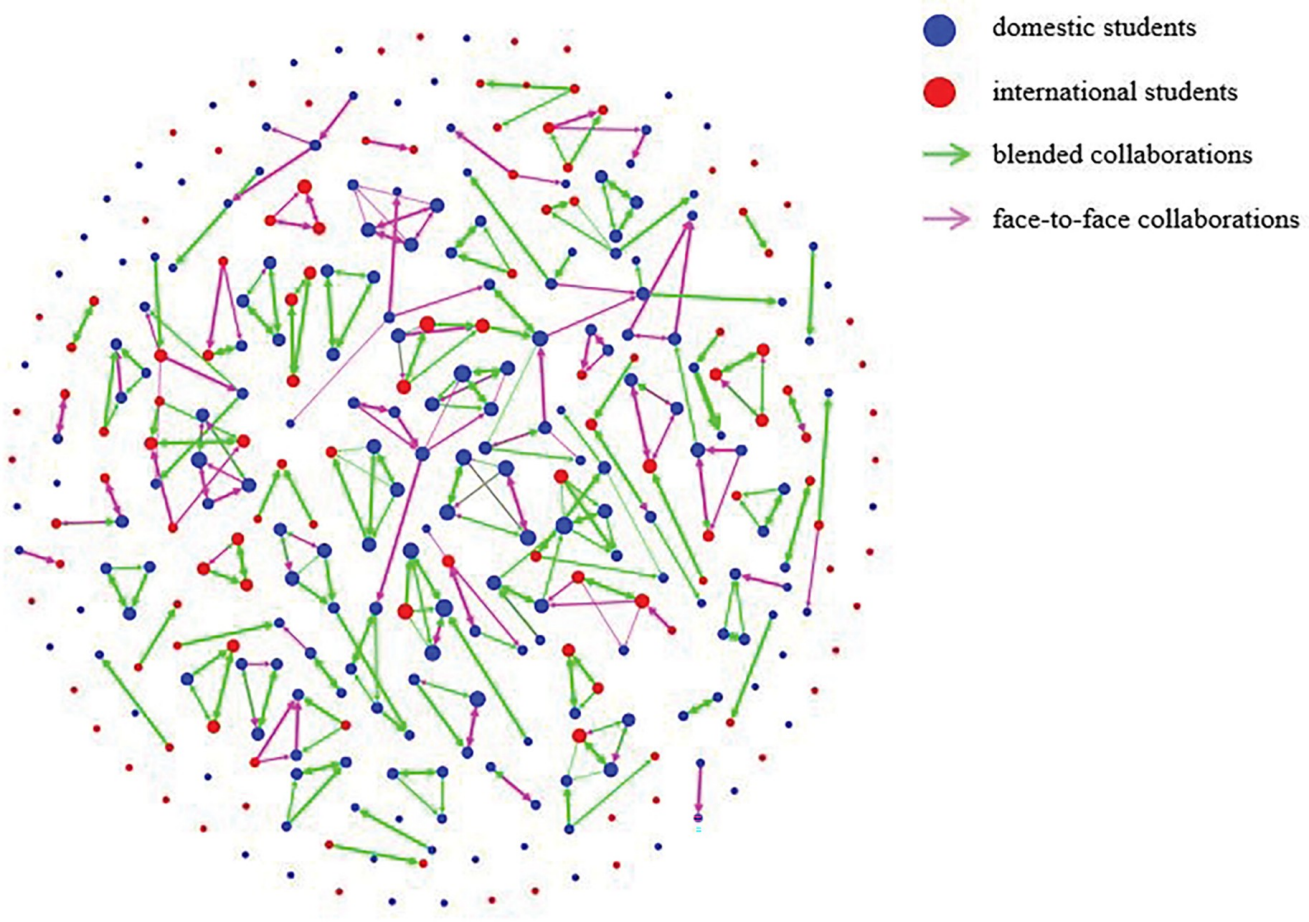
Visualization of the collaboration network.

Fig 1 provides the visualization of the collaboration network of this blended course. Table 2 shows the results of the two-sample *z*-tests of proportions of different types of collaborators between domestic and international students. Domestic students had a significantly lower proportion of working alone students than international students had: *z* = 2.70, *p* < .01. They also had a significantly lower proportion of students who only initiated collaborations than for international students. However, domestic students had a significantly higher proportion of students who both initiated collaborations and were nominated as collaborators than international students: *z* = 2.90, *p* < .01.

**Table 2:** Comparison of proportions of types of collaborators between domestic and international students.

Types of collaborators	Domestic (*n* = 193)		International (*n* = 120)		*z*	*p*
	*n*	%	*n*	%		
Alone	37	19.17%	39	32.50%	2.70	**.00**
Only initiating	16	8.29%	23	19.17%	2.90	**.00**
Only being nominated	38	19.69%	15	12.50%	1.60	.11
Both initiating and being nominated	102	52.85%	43	35.83%	2.90	**.00**

As all the SNA centrality measures were 0 for the students who did not report any collaboration, they were excluded from the analysis for the comparison of the SNA centrality measures between domestic and international students. The exclusion resulted 156 domestic and 81 international students for comparison, which are displayed in Table 3.

**Table 3:** Comparison of the SNA centrality measures between domestic and international students.

SNA metrics	Domestic (*n* = 156)		International (*n* = 81)			*F*	*p*	η^2^
	*M*	*SD*	*M*	*SD*				
Degree	3.08	1.57	2.64		1.43	4.34	**.04**	.02
In-degree	1.59	0.91	1.22		0.97	8.31	**.00**	.03
Out-degree	1.49	1.07	1.42		0.92	0.23	.63	.00
Closeness	0.67	0.42	0.72		0.39	1.02	.31	.00
Betweenness	1.44	5.13	0.59		2.87	1.92	.17	.01
Eigenvector	0.20	0.22	0.12		0.16	7.17	**.01**	.03
Local clustering coefficient	0.55	0.45	0.52		0.47	0.37	.55	.00

Table 3 shows that domestic and international students differed significantly on degree: *F* (1, 235) = 4.34, *p* < .05, η^2^ = .02; in-degree: *F* (1, 235) = 8.31, *p* < .01, η^2^ = .03; and eigenvector: *F* (1, 235) = 7.17, *p* < .05, η^2^ = .03. To be more specific, domestic students had higher degree than international students, suggesting that on average domestic students collaborated more than international students. Domestic students also had higher in-degree than international students, indicating that domestic students were nominated as collaborators more than international students were. Domestic students had a higher eigenvector centrality than international students, implying that domestic students tended to collaborate directly with those who were also had more collaborations in the network.

### 3.4 Comparison of the material elements of the learning experience between domestic and international students (results for research question 3)

The results of the comparison of students’ interactions with the online learning activities between domestic and international are presented in Table 4. The results show that international students interacted significantly more frequently with the dashboard: *F* (1, 311) = 4.39, *p* < .05, η^2^ = .02; and the online course reading: *F* (1, 311) = 5.08, *p* < .05, η^2^ = .02. International students also had a significantly higher proportion of pausing in the interactions with videos: *F* (1, 311) = 5.27, *p* < .02, η^2^ = .02; a higher proportion of multiple choice questions with showing the answer: *F* (1, 311) = 8.47, *p* < .01, η^2^ = .04; and multiple choice questions in videos with showing the answer: *F* (1, 311) = 15.09, *p* < .01, η^2^ = .07; than domestics students. But domestic students had a higher proportion of correctly answered multiple choice questions in videos: *F* (1, 311) = 8.00, *p* < .05, η^2^ = .04.

**Table 4:** Comparison of the material elements between domestic and international students.

Variables	Domestic (*n* = 193)		International (*n* = 120)			*F*	*p*	η^2^
	*M*	*SD*	*M*	*SD*				
Dashboard	398.06	186.15	454.09		211.89	4.39	**.04**	.02
Course readings	820.12	350.15	940.10		454.53	5.08	**.03**	.02
V_PL (%)	.36	.12	.34		.12	2.01	.16	.01
V_PA (%)	.54	.12	.58		.14	5.27	**.02**	.02
V_RL (%)	.10	.10	.08		.05	2.03	.16	.01
MCQ_C (%)	.56	.12	.53		.11	4.05	.05	.02
MCQ_I (%)	.33	.09	.32		.08	0.77	.38	.00
MCQ_S (%)	.11	.10	.15		.11	8.47	**.00**	.04
VMCQ_C (%)	.53	.12	.48		.13	8.00	**.01**	.04
VMCQ_I (%)	.36	.10	.35		.09	0.87	.35	.00
VMCQ_S (%)	.11	.10	.17		.13	15.09	**.00**	.07

*Notes*: V_PL = playing videos, V_PA = pausing videos, V_RL = reloading videos, MCQ_C = correctly answered multiple choice questions, MCQ_I = incorrectly answered multiple choice questions, MCQ_S = multiple choice questions with showing the answer, VMCQ_C = correctly answered multiple choice questions in videos, VMCQ_I = incorrectly answered multiple choice questions in videos, and VMCQ_S = multiple choice questions in videos with showing the answer.

## 4. Discussion

As blended courses has increasingly been utilized in higher education [34], more and more research has been conducted to examine students’ learning experience in blended courses [5–8]. However, there is a dearth of studies which compared domestic and international students’ learning experience in blended courses and they also produced inconsistent findings [10, 11]. Hence, the current study was designed to compare domestic and international students’ learning experience in blended courses in order to uncover actionable knowledge that can be used to improve the experience of both groups. Australia was selected as the context of the study because of its ability to attract international students [2]. Furthermore, the study investigated aspects across cognitive, social, and material elements of students’ learning experience as well as their academic achievement, which was more comprehensive than previous research [10, 11].

The findings of the study offer some interesting insights, which can help improve the experience of domestic and international students’ learning in blended courses. In our study, international students perceived the online part of their learning experience to be well-integrated with the learning activities and tasks in the overall course, and they valued the online contributions of the other students in the course. These findings were consistent with Dang et al.’s study that their international students also held more positive perceptions towards the blended course than domestic peers [10]. Our results, however, somewhat contradicts Chew’s findings that international students negatively perceived the online part of the blended courses [11]. Chew attributed international students’ negative perceptions to their lack of prior online learning experience as he reasoned that his participants were only in their first two years of their university studies. However, in both our study and Dong et al.’s study, the international students were first-year students. This seems to suggest that international students’ negative perceptions towards blended courses might not be necessarily due to their lack of experience with online learning. Clearly further investigation into the issue of whether international students’ perceptions of blended courses are related to their prior learning experience is required.

The self-reported differences on the students’ perceptions of their blended learning environment seem to be consistent with the results of the observational evidence. It appeared that international students perceived the integration between the face-to-face and the online learning more positively, which aligned with the observational results that they read online and used the dashboard more frequently than domestic students.

The consistency between the self-reported and observational data is also evident in that international students reported adopting more surface approaches to inquiry on the one hand; on the other hand, they also selected the showing the answer option more frequently than domestic students when they undertook the multiple choice questions and the multiple choice questions in videos. As surface approaches to learning primarily focus on reproducing knowledge and being dependent, the observation of international students’ reliance on the answers provided in the online system rather than solving these questions and problems on their own was consistent with their reported approaches. International students’ frequent reliance on the ready-made answers could also partially explain why they scored significantly lower in the final close-book examination than their domestic peers.

Another notable difference is significant more frequent pauses experienced by international students than domestic students when viewing video contents online. The reason for the frequent stop could be possibly caused by international students’ English proficiency, as past research consistently reported that language barrier was one of the common challenges faced by international students, especially in the first two years of their studies, as the case of our participants [35–37]. These investigations also revealed that international students were especially weaker in listening and speaking skills when they studied in a foreign language [38], hence, might affect their abilities to continuously engage with video study materials. However, this interpretation should be further investigated by directly asking international students to explain the frequent pauses when they viewed the online videos.

With regard to the comparison of the social aspects of the learning experience, the SNA results suggest that domestic students had better collaborative experience than international students. Specifically, a much lower proportion of domestic students reported working alone, and on average domestic students had more collaborations (as reflected by the degree centrality measure). We also found that a much higher proportion of domestic students were those who both initiated collaborations at the same time were nominated as collaborators; whereas a much higher proportion of international students only initiated collaborations but were not nominated as collaborators.

Such results seem to corroborate with previous findings that international students tend to be marginalized and ignored in collaborations and group work [39, 40]. Research has indicated that domestic students hold negative attitudes towards working in multicultural groups because they are concerned about international students’ language proficiencies, academic competencies, and cultural knowledge [41, 42]. Some domestic students also believe that having multicultural collaborations will generate negative impacts on the marks of the group assignments, and hence their final academic results [43]. For instance, Burdett reported that domestic students in an Australian university had negative comments about international students and believed that international students did not contribute to the group work, caused more time for the group work to be completed, and increased their workload and responsibilities [44]. Once again, some qualitative interviews with domestic students should be conducted in future studies to find out if this caused the low quality of collaborative experience of international students.

### 4.1 Practical implications

The results of our study offer some useful suggestions for teaching practice, especially for teaching classes with a large proportion of international students. Our study shows that across the cognitive, social, and materials aspects of students’ learning experience, international students particularly need help to improve their collaborative learning experience.

Radloff points out that effective collaborative learning between domestic and international students can be used to facilitate interaction and integration of multicultural groups and to increase social cohesion in learning [45]. To encourage such multicultural collaborative learning experience between domestic and international students, teachers may pre-assign groups by mixing domestic and international students. To avoid domestic students’ concern about undertaking more workload to obtain good performance in the group assignments, individual assessment strategies should also be included as part of the group assignment marks. For instance, teacher can ask students to write reflective journals about their collaborative experiences and their self-evaluation of their contributions to the group work, so that individual performance in the collaborative learning tasks and activities can be evaluated and counted towards the group assignment scores.

Teachers may also recommend some appropriate workshops offered by the university to international students for them to enhance their intercultural communication strategies and to better understand cultural differences and social rules [46]. Moreover, at the beginning of the course, appropriate class time should be allocated for students to discuss and share their opinions about effective collaborations so that mutual understanding can be reached before collaborations in order to achieve more desirable outcomes [39].

### 4.2 Methodological implications

One of the strengths of the study is its ability to reflect on different aspects of the learning experience of domestic and international students rather than focusing on merely one aspect. This helps reveal specific aspect(s) of the differences between domestic and international students so that strategies which target related issues impeding learning can be implemented by teachers. The ecological perspective adopted in this study helps uncover related evidence of variations in students’ learning experience by using multiple methodologies from SAL, SNA, and learning analytics research. Each method offers a useful perspective on variations in one aspect. When combined, the analyses provide a deeper insight into the differences between domestic and international groups than the use of any single methodology.

For example, in this study, we found that the self-reported evidence of the more positive perceptions of the blended learning environment by international students were consistent with the observational evidence of their more frequent interactions with a number of online learning activities. The methodological approach of combining the self-report and observable data offers promise for it to be used in future studies to investigate the complexity of the learning experience in blended courses.

### 4.3 Limitations of the study

Limitations of the study should be pointed out to keep in mind when interpreting the results. While the findings are illuminative, the research context is just one course. Many more comparative studies like this should be undertaken in order to assess the robustness of the claims. Furthermore, the research design is quantitative, which partially limits the depth of description of reasons behind the differences. To more deeply understand the differences between domestic and international students’ learning experiences, qualitative research methodologies, such as focused groups or semi-structured interviews, should be employed to supplement the quantitative findings in future research programs.

## 5. Conclusion

Adopting an ecological perspective, the current study compared the cognitive, social, and material aspects of university students’ learning experience in blended courses between Australian domestic and international students. The results found differences on all the three aspects between the two groups of students. Similar to previous findings, the current study also showed that international students particularly need help to improve their collaborative learning experience. Possible strategies, such as mixing international students with domestic students in group work and improving international students’ intercultural communication competence through well-designed workshops, may help to enhance international students’ experience in collaborative learning.
